# The Crossroads of Geriatric Cardiology and Cardio-Oncology

**DOI:** 10.1007/s13670-015-0147-4

**Published:** 2015-09-16

**Authors:** Kim-Lien Nguyen, Rami Alrezk, Pejman G. Mansourian, Arash Naeim, Matthew B. Rettig, Cathy C. Lee

**Affiliations:** VA Greater Los Angeles Healthcare System, 11301 Wilshire Blvd, MC 111E, Los Angeles, CA 90073 USA; Division of Cardiology, David Geffen School of Medicine at UCLA, Los Angeles, CA 90073 USA; Division of Geriatrics, David Geffen School of Medicine at UCLA, Los Angeles, CA USA; GRECC, VA Greater Los Angeles Healthcare System, Los Angeles, CA USA; Department of Urology, David Geffen School of Medicine at UCLA, Los Angeles, CA USA; Division of Hematology-Oncology, David Geffen School of Medicine at UCLA, Los Angeles, CA USA

**Keywords:** Geriatric cardiology, Geriatric oncology, Cardio-oncology, Chemotherapy-induced cardiomyopathy

## Abstract

Cancer and cardiovascular disease (CVD) are two major causes of mortality in older adults. With improved survival and outcomes from cancer and CVD, the role of the geriatrician is evolving. Geriatricians provide key skills to facilitate patient-centered and value-based care in the growing older population of cancer patients (and survivors). Cancer treatment in older adults is particularly injurious with respect to complications stemming from cancer therapy and as well as to CVD related to cancer therapy in the context of physiologic aging. To best meet their natural potential as caregiving leaders, geriatricians must hone skills and insights pertaining to oncologic and cardiovascular care, insights that can inform and enhance key management expertise. In this paper, we will review common chemotherapy and radiation-induced cardiovascular complications, screening recommendations, and advance the concept of a geriatric, cardiology, and oncology collaboration. We assert that geriatricians are well suited to a leadership role in the care of older cardio-oncology patients and in the education of primary care physicians and subspecialists on geriatric principles.

## Introduction

Cancer and heart disease are the leading cause of death in patients older than 60 years of age [1]. While cancer is the number one cause of mortality in patients between 60 and 79 years, heart disease is most common in people aged 80 and older [1]. Therefore, the role of geriatricians in these specific subspecialties is of particular significance. Geriatric cardiology and geriatric oncology have long been respective partners because advances in cancer and cardiovascular (CV) care have increased life expectancy. With the increasing need to address CV consequences of chemotherapy and/or radiation therapy, the field of “cardio-oncology” or “onco-cardiology” faces renewed interest. Because cancer and heart disease are pathology related to the aging process, a close partnership between geriatric cardiology and cardio-oncology is also natural, and geriatricians have an important opportunity to develop as clinical leaders at this emerging crossroad. In many cases, the goals of management are not solely focused on survival but also on other markers such as quality of life, functional status, and individual patient preferences.

We present a case study to demonstrate tensions within this burgeoning discipline: a fully functional and active 69-year-old Caucasian female with a history of chronic anemia, hypertension, and dyslipidemia was admitted for hip fracture following a mechanical fall. During the evaluation of her fall, she was diagnosed with multiple myeloma as the underlying etiology of her fracture. With daily intensive physical therapy, she made gradual and steady improvement in her functional status. At the oncology visit, she was considered to be “high risk” based on genetic testing and was initiated on induction therapy with four cycles of bortezomib, lenalidomide, and low-dose dexamethasone. Subsequently, she was admitted with new onset shortness of breath and leg swelling and found to have new onset congestive heart failure (CHF) and extensive right iliac vein thrombosis. Echocardiography showed a left ventricular ejection fraction (LVEF) of 20–25 % despite a normal pre-chemo echo. Cardiology was consulted. Left heart catheterization showed non-obstructive coronary artery disease (CAD), and she was started on a cardiomyopathy regimen. Potential placement of an implantable cardioverter defibrillator (ICD) if her LVEF remained low after optimal medical therapy was discussed.

The patient’s sequelae of CHF and venous thrombosis were complications of bortezomib [2] and lenalidomide [3], respectively. Although the development of cardiomyopathy might have been unavoidable, how might we approach her case if she were a frail vs functional 69-year-old? Given her age and risk factors for CAD, should she have undergone a CV evaluation prior to the therapy initiation? This case is an example of the current practice model whereby management of CV complications is reactionary. Although the patient was managed accordingly, her care could have been enhanced with proactive involvement of cardiology during the evaluation for chemotherapy. In geriatric patients, particularly those who have limited functional status, complications from chemotherapy and/or radiation may be insidious and present themselves much later.

Excellent reviews on the CV effects of chemotherapy and/or radiation therapy [4••, 5••, 6–12] as well as malignancies common to older adults and decision-making strategies [13, 14, 15••, 16, 17] have been published. Barac et al. have also outlined a roadmap for taking cardio-oncology to the next level in clinical care, research, education/training, and in establishing collaborative networks [18••]. In this review, we aim to provide a basic foundation for understanding chemotherapy and/or radiation-induced CV complications, discuss a framework for geriatric–cardiology–oncology (geri-cardio-onc) collaborations, and advocate for a paradigm shift to enable geriatricians to lead the conversation both in advocating for care and in educating other physicians about geriatric principles.

## Cardiotoxicity and Guideline-Based Screening

Cancer therapeutics including traditional chemotherapy, targeted therapy, radiotherapy, and hormonal therapy all have short- and long-term systemic effects, often involving multiple organs. However, CV toxicities have been most concerning and can result in irreversible CV damage or reversible cardiac dysfunction. These adverse events may be related to direct effects on myocytes or indirect effects on the CV system, which in turn may increase the risk of CV disease [9]. Stuter and Ewer [19] suggested a classification system for identifying chemotherapies causing irreversible (type I cardiotoxicity, associated with cell loss, anthracyclines and mitoxantrone) and reversible cellular dysfunction (type II cardiotoxicity, dysfunction of mitochondria and protein structures, biologic agents) [20]. Despite limitations of this classification system (such as irreversible damage precipitated by a type II agent in a patient with pre-existing CV disease), the concept is foundational in deciding whether or not to proceed with cancer therapy. A spectrum of chemotherapy and/or radiation-induced CV complications exists: arrhythmias, cardiomyopathy and left ventricular dysfunction (LVD), valvular and pericardial disease, ischemic disease, as well as vascular disease. However, LVD and cardiomyopathy have received significant attention in recent years due to their long-term implications.

A consensus definition for cardiotoxicity is still lacking. The Cardiac Review and Evaluation Committee defines LV dysfunction as “(1) a decrease in LVEF that was either global or more severe in the septum, (2) symptoms of congestive heart failure, (3) signs of CHF (S3 gallop, tachycardia, or both), and (4) decline in LVEF of at least 5 % to less than 55 % with signs or symptoms of heart failure or a decline of LVEF by at least 10 % to below 55 % without signs or symptoms” (of heart failure) [5••]. Other definitions include a larger change in LVEF to less than 50 %. According to recommendations by the American Society of Echocardiography (ASE) and the European Association of CV Imaging (EACVI) [21••], LVD may be defined as a decrease of greater than 10 % to an LVEF <53 % by 2D echo or a decrease of >15 % in 2D-derived global longitudinal strain (GLS) from baseline echo. At baseline, LVD is characterized as 2D LVEF <53 % with confirmation by cardiac MRI or 2D GLS that is less than the lower limits of normal (which is vendor-dependent). In addition to a decrease in LVEF and the symptomatic vs asymptomatic nature, another dimension warranting inclusion is reversibility: “reversible = improvement to within 5 percentage points of baseline; partially reversible = improvement by 10 points but remaining within 5 percentage points of baseline; irreversible = remaining within 10 percentage points of the nadir; and indeterminate = re-evaluation not available” [21••]. The timeframe for re-assessing reversibility varies depending on the specific chemotherapeutic agent but can be generalized based on recommendations summarized in Table 1.

**Table 1:** General algorithms for screening and monitoring of chemoradiation-induced cardiovascular complications [21••, 27••, 59••]

Chemotherapeutics—anthracycline-based therapy (type I injury)^b^ [5••]
• Baseline cardiac evaluation and echocardiogram
• Troponin evaluation at each cycle
Troponin positive
▪ Enalapril × 1 year; echo at completion of chemo, then at 3–6–9–12 months post-chemo in the first year
▪ Echo every 6 months after the first year × 5 years
Troponin negative
▪ Echo at 12 months, then echo every year thereafter
• No troponin evaluation during chemotherapy
▪ Echo at end of chemotherapy and at 3–6-9–12 months post-chemo in the first year, then annually
▪ If LVD^a^ develops, then treatment with ACEI + BB + clinical evaluation and follow-up
Chemotherapeutics—trastuzumab therapy (type II injury)^b^ [5••]
• LVEF ≥50 % → initiate treatment
• LVEF <40 % → hold treatment and repeat echo in 3 weeks
• LVEF between 40 and 50 %
• LVEF >10 % points below baseline
▪ Hold treatment, repeat echo in 3 weeks
▪ If repeat LVEF <40 %, stop treatment
▪ If repeat LVEF ≥45 % or remains 40–50 %, then resume treatment
LVEF <10 % points below baseline → continue treatment
Radiotherapy [59••]
• Baseline pre-radiation echocardiogram
• Yearly focused clinical exam searching for signs and symptoms of cardiovascular disease, screen for modifiable risk factors and treat
• Diagnostic evaluation if signs/symptoms of cardiovascular disease
• Asymptomatic
Screening echo at 5 years for high risk patients and 10 years for others
Functional non-invasive stress test for detection of ischemic heart disease 5 to 10 years after exposure in high risk patients
Reassess every 5 years for need of echo and/or stress testing

^a^Consensus definition for left ventricular dysfunction (LVD) is lacking but is defined as a decrease of greater than 10 % to an LVEF <53 % by 2D echo or a decrease of >15 % in 2D-derived global longitudinal strain (GLS) from baseline echo [21••]. At baseline, LVD is characterized as 2D LVEF <53 % with confirmation by cardiac MRI or 2D GLS less than the lower limits of normal. Repeat echo should be performed 2–3 weeks after the baseline study showing a decrease in LVEF

^b^The American Society of Echocardiography and the European Association of Cardiovascular Imaging recommend cardiology consultation in both type I and type II injury when LVEF ≤53 % and suggest increased sensitivity and specificity for detection of subclinical disease when LVEF is used in concert with GLS measurements during the initiation and monitoring of cardiotoxicity [21••]

For many physicians and patients, acute or short-term effects of chemotherapy and/or radiation therapy tend to be at the forefront of their concerns. However, potential late effects, which can present decades later, are often insidious. Therefore, in certain populations, such as older adult survivors of cancer, vigilance, and awareness may assist in earlier diagnosis.

### Cardiomyopathy and Vascular Effects

Major groups of chemotherapeutic drugs that can cause cardiotoxicity, the time to presentation, their proposed mechanisms, and strategies for mitigation are outlined in Table 2. Of these agents, anthracyclines are the most widely studied and represent the most effective class of drugs for hematologic and solid organ malignancies [22, 23] such as sarcomas, lymphomas, leukemias, and breast cancer. However, their use is limited by potential LVD and heart failure. Anthracycline-induced cardiotoxic effects are dose dependent, and those who develop late cardiotoxicity have a high mortality [24]. Risk factors include cumulative dose, bolus administration, high single dose, prior radiotherapy, simultaneous use of other cardiotoxic agents, female gender, bimodal age distribution (very young and very old), existing CV disease, elevation of cardiac biomarker during and after cancer treatment, as well as time since completion of cancer therapy. Nonspecific electrocardiographic changes such as ST-T wave abnormalities have been observed in 20 to 30 % of patients undergoing therapy with anthracyclines [25] and may herald early manifestations of anthracycline cardiotoxicity but are often not acted upon due to lack of other clinical manifestations. Important in the determination of risk is the predisposition to having cardiotoxic effects. This predisposition is thought to be multifactorial and reflects a combination of genetic, environmental, and existing medical comorbid conditions [26]. Much work in the area of -omics (genomic, proteomic, metabolomics) and risk modeling is needed to provide personalized risk stratification. Further, significant vigilance is needed during and after therapy, particularly in the older population who often have a high burden of underlying CVD. Although observational and clinical data exist to support increases in risk of anthracycline-induced cardiotoxicity with age (>65 years for anthracyclines [27••], >80 years for trastuzumab [28]), it is less clear whether there is an association of cardiotoxicity with age for newer therapies, but the latter may be reflective of an under-representation of adults older than 65 years and especially those >80 years, in clinical trials [29].

**Table 2:** Cardiovascular toxicity of common chemotherapy agents [4••, 5••, 11]

Class	Cardiovascular toxicity	Time to presentation	Mechanism	Mitigation
Anthracyclines Doxorubicin Daunorubicin Epirubicin Idarubicin Mitoxantrone	LV dysfunction CHF Pericarditis Myocarditis	Weeks to decades	Oxidative stress Mitochondrial dysfunction Direct DNA damage Topoisomerase 2b	•Dose modulation •Continuous infusion •Liposomal formulations •Beta blockers •ACE inhibitors •Dexrazoxane
Alkylating agents Cyclophosphamide	Ischemia, hypertension, CHF	Ischemia, hypertension, CHF		
	Pericarditis, myocarditis, CHF, hemorrhagic myopericarditis	Acute (days to weeks)	Direct oxidative cardiac injury Not associated with cumulative dose	Lowering the dose
Antimetabolites Capecitabine 5-Fluorouracil	Ischemia, cardiogenic shock, vasospasm, CHF, arrhythmia, angina	At time of administration	Vasospasm Arteritis Direct myocardial toxicity	•Nitrates, CCB •Termination of treatment (reversible), retreatment after symptom improve •IV bolus regimen, lower dose regimen •Anti-anginal treatment •Prophylactic coronary vasodilator (limit efficacy)
Antimicrotubules Docetaxel Paclitaxel	Sinus bradycardia, heart block, ventricular tachycardia, Hypotension CHF	During administration	Arrhythmic mechanisms unknown; can potentiate AC cardiotoxic effects	•Pre-treatment with corticosteroid, H_1_ and H_2_ blocker agents •Dose adjustment and limiting dose of anthracycline •ECG monitoring •Continuous infusion with infusion monitoring for hypersensitivity reaction
Targeted agents (monoclonal antibody-based TK and small TKIs)				
Bevacizumab	Hypertension, CHF, DVT	Vascular (HTN, PH)—days to months Ventricular dysfunction—days to weeks Fluid retention—days to weeks QT prolongation—during administration	VEGF inhibition Endothelial cell dysfunction	•Anti-hypertensives •Smooth muscle relaxants •Temporary interruption •Diuretics •Avoid other QT prolonging agents; termination if QTc >470 ms
Cetuximab	Hypotension		Anaphylaxis and hypersensitivity reaction Electrolytes imbalance	
Rituximab	Hypotension, angioedema, arrhythmias		Anaphylaxis and hypersensitivity reaction	
Trastuzumab	CHF, LV Dysfunction		anti-Erb2 (transmembrane receptors)	
Imatinib	QT prolongation		Significant mitochondrial dysfunction	
Sorafenib	LV dysfunction		RAF1 inhibition Myocyte apoptosis	
Proteasome inhibitors Bortezomib Carfilzomib	LV dysfunction, CHF	Can be acute—duration unknown	Induces ER stress	•Termination of treatment (reversible) •Dose modification based upon cardiac toxicity •Caution in patient with history of syncope •Avoid of dehydration
Radiotherapy	CAD, pericarditis, Myocarditis, CHF Valvular disease Conduction disease Vascular disease	Months to decades	Generation of reactive oxygen species Endothelial damage Arteritis Cardiac fibrosis	Shielding and fractionated dosing

*AC* anthracycline, *CAD* coronary artery disease, *CCB* calcium channel blocker, *CHF* congestive heart failure, *DVT* deep venous thrombosis, *ER* endoplasmic reticulum, *HTN* hypertension, *LV* left ventricular dysfunction, *NO* nitric oxide, *PH* pulmonary hypertension, *TK* tyrosine kinase, *TKI* tyrosine kinase inhibitors, *VEGF* vascular endothelial growth factor

A second class of frequently used cardiotoxic agents are targeted therapies including monoclonal antibody-based tyrosine kinases (bevacizumab, trastuzumab) and small molecule tyrosine kinase inhibitors (sorafenib, sunitinib, lapatinib) [30–32]. Targeted therapies are often used in breast, lung, colorectal, and renal carcinomas. Hypertension is a common adverse event whose mechanism is not well understood but has been attributed to the inhibition of vascular endothelial growth factor (VEGF) [33, 34], which can result in decreased microvascular density. Thromboembolic events are infrequent but have also been implicated with anti-VEGF agents [7]. LV dysfunction also occurs but less common. Of the targeted agents, trastuzumab has been found to have a higher than expected incidence of CHF and LVD [35, 36] and can pose higher risk of LVD in those previously treated with anthracyclines or those undergoing concurrent therapy with anthracyclines.

### Ischemic Heart Disease

Cardiac ischemia is a known complication of many chemotherapeutic drugs but is an unusual occurrence and more often associated with cytotoxic and targeted therapy. Progression of or acceleration of ischemic disease is more common with radiation therapy. Agents such as bleomycin, etoposide, cisplatin [37–40], 5-fluorouracil (FU) [41–44], as well as newer anti-VEGF agents [45, 46] have been implicated in the development of myocardial ischemia including myocardial infarction. The pathogenesis of 5-FU-mediated cardiac ischemia appears to involve endothelium-independent vasoconstriction and coronary vasospasm [47]. Although coronary vasospasm appears to be central in the development of myocardial ischemia from 5-FU, arterial thromboembolism may also be implicated [45, 46]. A post hoc analysis by Scappaticci et al. showed a higher risk of arterial thromboembolism in patients treated with conventional chemotherapy and bevacizumab as compared to patients treated with chemotherapy alone (3.8 vs 1.7 % (95 % CI = 0.7 to 3.7 %) [48]. As newer targeted therapies become more widely used, those with anti-VEGF or anti-angiogenesis properties require close attention, particularly when used in patients with risk factors for CAD or known CAD. For older men with prostate cancer undergoing treatment with androgen deprivation therapy (ADT), the verdict remains inconclusive as data on the association of ADT with CVD have been inconsistent. Although there are reports of an increased risk of heart disease including CAD, acute ischemia, and sudden cardiac death [49–54], there are also other studies showing no increased risk of CV morbidity and mortality [55–57].

### Radiation-Induced Injury

A large body of literature supports CV injury following chest radiotherapy [5••]. However, the patient population that is most affected by radiotherapy-induced CV injury is adult cancer survivors treated with radiotherapy at a relatively young age and therefore has a longer time horizon to develop late cardiac effects. These typically include those with Hodgkin’s lymphoma, early stage breast cancer, lung, and esophageal cancer. Mediastinal radiotherapy may lead to disease of the coronaries, valves, myocardium, pericardium, and conduction system [58]. Risk factors for radiation-induced CV injury include radiation dose >30–35 Gy or dose per fraction >2 Gy, anterior or left chest irradiation, large volume of cardiac exposure, younger age at exposure, longer time since exposure, combination therapy with cytotoxic chemotherapy, hormonal therapy, anthracyclines, or trastuzumab, known CV disease, as well as risk factors for cardiac disease (diabetes, hypertension, dyslipidemia, obesity, smoking) [59••]. For those with Hodgkin’s lymphoma, the time horizon for risk of fatal CV events is 2–7 years post-radiotherapy, while for those with left-sided breast cancer, the range is 1.0–2.2 years [5••].

### Device Therapy

Some patients face acute LVD during treatment, while others may present with LVD as a late effect. If LVD remains ≤35 % after treatment with optimal cardiomyopathy medications and if there is greater than one year of expected survival, patients are eligible for ICD therapy as primary prevention of sudden cardiac death [60]. Although studies have shown improved survival in patients with ICD compared to optimal medical therapy alone [61, 62] and there is improved quality of life in those who have not experienced shock therapy [63, 64], these data are not specific to those who have LVD secondary to chemotherapeutics. Further, chemotherapy and/or radiation therapy may cause conduction disease or progression of underlying conduction disease, resulting in the need for pacemaker therapy. In discussing device therapy, particularly in the older cancer patient, it is important to stress that ICD therapy can be easily deactivated at any time to prevent shocks (i.e., when shock therapy becomes dyssynchronous with the patient’s goals of care). Deactivation of pacemakers on the other hand may rarely result in immediate death for those who become pacemaker dependent. Importantly, pacemaker deactivation can induce a rapid progression of symptoms such as fatigue, dizziness, or shortness of breath [65]. In deciding which patients are appropriate and may benefit from advanced device therapy, one must consider the patient’s overall cancer prognosis, social support risk of complications such as bleeding and infection, as well as the patient’s preferences.

### Guideline-Based Screening and Monitoring

Surveillance and monitoring consensus statements by the European Society for Medical Oncology (ESMO) [5••] and imaging societies [21••, 59••] have been published. The National Comprehensive Cancer Network (NCCN) [66] and Society for Geriatric Oncology [27••] (SIOG) have also released publications on the care of the older cancer patient—both of which cover CV complications and management. NCCN also provides age-specific guidelines on their website with recommendations outlining treatment of anthracycline-related cardiotoxicity. Table 1 summarizes general algorithms for monitoring and management. Publications by SIOG and NCCN [27••, 66] address the older population specifically, while the joint scientific statement by the ASE and the EACVI [21••] address monitoring in adults of all ages. The statement by Lancellotti et al. provides recommendations for adults treated with radiotherapy [59••]. Despite recent scientific statements, a paucity of data exists to help form strong evidence-based recommendations in the cardio-oncologic care of the older, medically complex patient. Dale et al. in their article on the interface of cancer and aging research [67] outlined selected clinical trials which enrolled older patients with cancer (12 in total—two trials in breast cancer, two lung cancer, two colorectal cancer, two acute myeloid leukemia, three prostate cancer, and two ovarian cancer). They noted several themes: (1) few clinical trials are designed specifically for older patients with cancer, (2) the actual proportion of older patients in clinical trials is not reflective of that in the general population, and (3) measures of functional/physiologic age are not routinely included in the study design. Often, older patients with known CV disease are also excluded. Further, it is difficult to know whether the CV complications observed in these trials reflect the true incidence and prevalence in the general older adult population as many older patients have subclinical cardiac disease that may manifest during the course of cancer therapy or be exacerbated by the treatment.

### Chronologic vs Physiologic Age and the “Fitness” of an Older Patient with CV Disease to Undergo Cancer Therapy

Chronologic age by itself is a poor predictor of outcomes including life expectancy, functional reserves, and risk of complications [68]. Risk factors for chemotherapy associated adverse events involve a combination of underlying comorbidities (renal insufficiency, lung disease, hearing or visual loss, gastrointestinal disease, neuropathy, osteoporosis, diabetes, anemia, known CHF), geriatric syndromes (functional dependence, decline in mobility, falls, dementia, delirium, depression, polypharmacy, nutritional deficiency), and socioeconomic factors (low income, lack of caregiver or transportation, poor living conditions, or lack of drug coverage) [66, 67, 69, 70]. Both oncologists and cardiologists are often called upon to assess an older patient’s “fitness” to undergo cancer therapy, particularly if the patient has known ischemic heart disease or LVD. Predictive models such as Chemotherapy Risk Assessment Scale for High-Age Patients (CRASH) [71] or Cancer and Aging Research Group (CARG) [70] are available for assessing chemotherapy risk and take into account the geriatric assessment in predicting toxicity. The Karnosky performance status, which is based on functional impairment, can also be used to predict risk of chemotoxicity. However, it has been shown to be less predictive than CARG, which takes into account additional factors such as tumor and treatment variables, laboratory values, and geriatric assessment questions [70]. These models and scores are more likely familiar to oncologists and some geriatricians but are likely less familiar to cardiologists or cardio-oncologists. However, the shared traits among these scores include the ability to walk a block, falls, and gait speed—all reflecting cardiac health.

Of the statements reviewed, those issued by ESMO [5••] are most explicit regarding a baseline, pre-chemotherapy evaluation. Note that level I–III reflects the strength of recommendation (I—there is general agreement that the recommendation is useful and effective, II—there is divergence in opinion whereby IIa indicates that the weight of evidence/opinion is in favor of usefulness and IIb indicates that the usefulness is less well established, III—there is general agreement that the recommendation is not effective and in some cases harmful), while letters A–C reflect the level of evidence (A—data derived from multiple randomized trials, B—data derived from single randomized or nonrandomized studies, C—consensus expert opinions). The recommendations for a baseline assessment include (1) assessment of CV risks including CAD and hypertension (level IA) based on prior cumulative doses of anthracyclines (level IA: doxorubin >500 mg/m^2^, liposomal doxorubicin >900 mg/m^2^; epirubicin >720 mg/m^2^; mitoxantrone >120 mg/m^2^, idarubicin >90 mg/m^2^); (2) LVEF assessment (level IA, if echocardiogram is performed, diastolic measurements and LV end-diastolic diameter should be noted); (3) 12-lead electrocardiogram (level IB); (4) biomarkers including troponin, brain natriuretic peptides, and neutrophil glucosaminidase-associated lipocalin for renal injury (level IIIB); (5) treatment optimization of existing cardiomyopathies and anti-ischemic regimen including revascularization if clinically appropriate (level IA); and (6) minimization of cardiotoxicity through the use of liposome-encapsulated doxorubin and cardioprotective agents such as dexrazoxane, beta blockers, ACE inhibitors, and aldosterone antagonists (level IIIB). No recommendation exists for routine performance of stress testing in the absence of ischemic symptoms or findings prior to chemotherapy.

## Geri-Cardio-Onc Collaboration and a Paradigm Shift

**Fig. 1:**
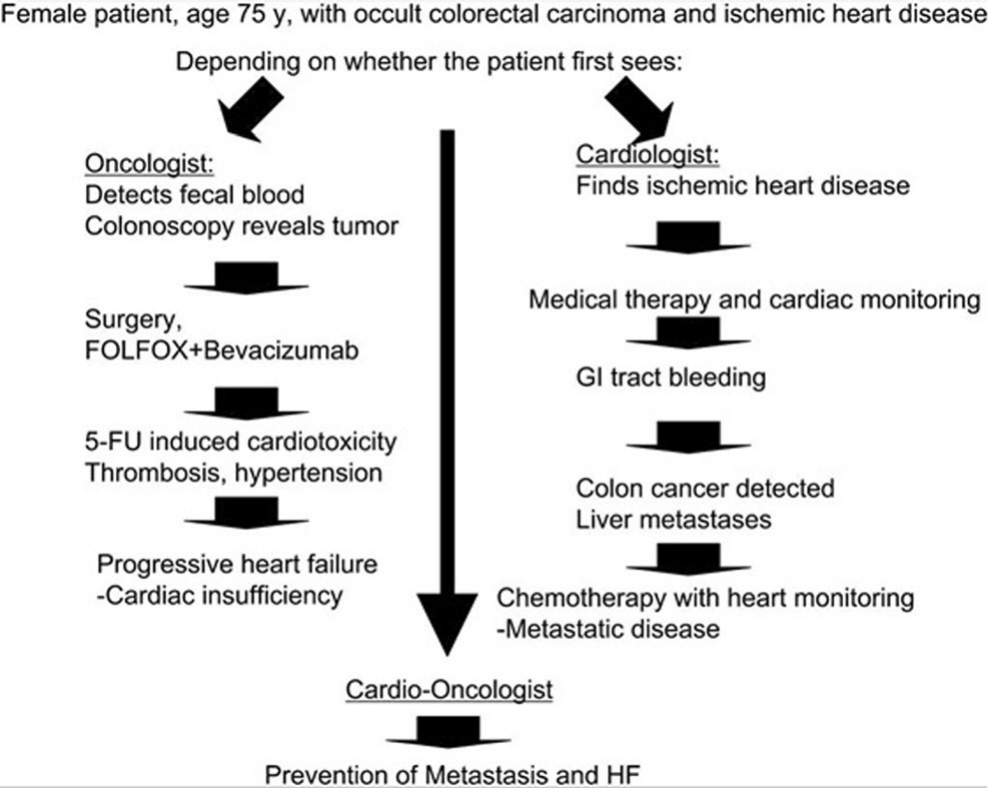
The “sliding doors” concept—an example of diverse outcomes based on first diagnosis [72]. The oncologist approaches the patient from a cancer perspective and proceeds with therapy accepting the risk of later cardiomyopathy diagnosis, while the cardiologist identifies the ischemic heart disease, proceeds with appropriate therapy, and later finds cancer from symptomatic bleeding secondary to anti-platelet therapy. From Albini et al. [72], by permission of Oxford University Press

Because those 65 and older are more likely to have multiple and complex medical diagnoses, having geriatricians assume leadership in this conversation helps to avoid the “sliding door” phenomenon [72]. In practices or institutions without geriatricians, having primary care physicians who are well versed in geriatric principles take the leading role can be also be effective. The “sliding door” concept was coined by Albini et al. and refers to the probability of a patient having diverse outcomes based on first subspecialty encounter, such as whether the patient was first seen by a cardiologist or an oncologist (Fig. 1). Once diagnosed with cancer, the patient’s care is shifted to the oncologist for primary management; similarly, if CV complications arise such as LVD, primary management is then shared between cardiology and oncology.

**Fig. 2:**
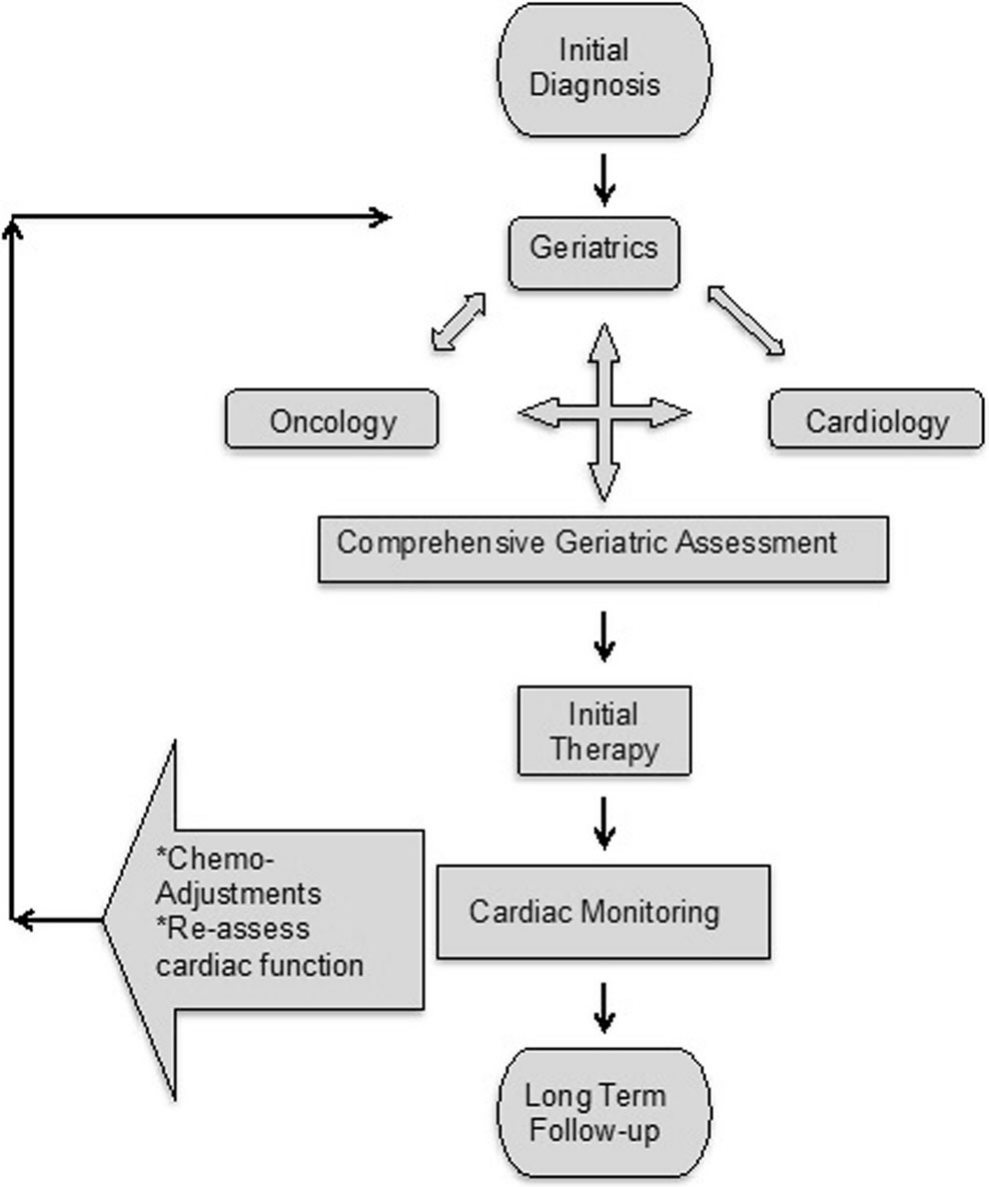
A triad approach for geriatric–cardiology–oncologic care. In this framework, the geriatrician (or primary physician well versed in geriatric principles) takes the leading role by ensuring that treatment options are discussed and presented in the context of the patient’s goals of care. Further, the geriatrician facilitates care by bringing to the forefront findings from the geriatric assessment and psychosocial dimensions that may affect treatment recommendations. Frequency of follow-up studies and monitoring of cardiovascular symptoms would rest with the geriatrician and co-managed with the assistance of both cardiology and oncology as needed

We would like to advocate for a collaborative patient-centered paradigm (Fig. 2) whereby there is greater dialogue throughout the course of care among three disciplines: geriatrics, oncology, and cardiology. This paradigm shift brings to the forefront a prior concept whereby the geriatrician serves as the patient’s primary advocate. Subspecialty expertise is sought, but the ultimate decision to proceed with subspecialty recommendations and subsequent screening and follow-up will rest with the geriatrician.

Although subspecialists are qualified to facilitate goals of care (GOC) discussions and assess the patient’s “fitness” to undergo chemotherapy and radiation treatment, geriatricians are better positioned to lead the conversation and address these issues from a holistic approach rather than an organ or disease-specific viewpoint. Detailed GOC discussions, which may include explanation of the possible mechanisms of death, are delicate and sometimes daunting matters. Often, complex issues regarding GOC are not discussed early enough or at all until a sudden catastrophic event or diagnosis occurs. Recent work by Narang et al. reported increased implementation of durable power of attorney assignment (DPOA) by cancer patients between 2010 and 2012, but this was not associated with end-of-life or GOC discussions [73]. Although there are limitations associated with proxy-reported research methodology, these findings support a communication gap between patient, surrogates, and clinicians [74].

Having advance directives in place is a starting point but is often insufficient when dealing with older adult patients with multi-comorbidities [75, 76]. Living wills often vary in detail, whereas DPOAs are not always capable or do not have prior knowledge of the patient’s preferences. GOC discussions require constant ongoing negotiations, particularly in cases of patients with cancer and heart disease whereby treatment of one carries significant complications for other comorbidities. Within the GOC discussion, dialogue on outcomes following cardiac arrest and cardiopulmonary resuscitation (CPR) merits specific attention. Although recent work by Chan et al. [77••] concluded that 60 % of adults age 65 years or older were alive at one year following in-hospital cardiac arrest and that the 3-year survival rate is similar to patients with heart failure, others argue that the relevant point of discussion should have centered on the finding that among older survivors of in-hospital cardiac arrest, less than 10 % are alive at one year [78•] while the rate of survival to discharge is 17 % across all ages [79•]. Glavan et al. [80•] have also pointed out that the majority of those of who receive in-hospital CPR die before discharge and that while CPR resulted in 27 % of patients achieving return of spontaneous circulation, the same percentage of patients did not survive to discharge [81]. In older patients with out-of-hospital cardiac arrest (OHCA), there is a 25 % survival to discharge from the intensive care unit, 22 % survival to hospital discharge, 20 % at 6 months, 19 % at 1 year, and 8 % at 5 year follow-up [82]. More recent data indicate that advanced age (>70 years) is an independent predictor of mortality in OHCA, albeit there was a small subset of those >90 years who had a survival rate of >10 % [83].

Glavan et al. [80•] have suggested that CPR decisions be based “on the patient being informed about both the likelihood of outcomes that are acceptable as well as the likelihood of outcomes that would not be acceptable.” This suggestion may prove more crucial in older adults with cancer and heart disease. Having at minimum, an annual appointment dedicated solely to the discussion of GOC with further re-negotiation each time a major transition in the treatment plan occurs would be especially helpful. If possible, these discussions should be detailed in progress notes and be made available to the entire medical team. While the treatment of cancer may not result in death, treatment complications may result in progression of heart disease, which may significantly alter the quality of life. Few patients consider the potential for a decrease in quality of life during the recovery period and many do not consider the unintended consequences of treatment when deciding on treatment options.

In the era of accountable care with medical homes, it is assumed that there is a sense of greater intimacy in the patient–primary physician relationship and awareness of psychosocial circumstances by the primary physician [85]—in this case, a geriatrician, whose experience and expertise in communicating with the older adult population may have immeasurable effects on patient outcomes and treatment preferences. Geriatricians are well-trained to manage patients with multiple chronic conditions in a systematic, routine, and patient-centered manner [84••]. Tools such as the comprehensive geriatric assessment [68], life expectancy scoring [69], and frailty assessment are common knowledge to the geriatrician. Cardiologists and oncologists may be less familiar with and often have limited to no training in routinely performing geriatric assessments and systematically evaluating for frailty. In addition, geriatricians can help cardiologists and oncologists improve the quality of care by proactively having GOC discussions with the patient and his/her DPOA and communicating the impact of dementia, depression, issues of nutritional deficiency, polypharmacy, as well as socioeconomic factors (low income, lack of caregiver or transportation, poor living conditions, etc.) [16, 17]. These issues often affect treatment options.

Although we have focused on geriatricians assuming a leading role, we recognize that there are unlikely sufficient geriatricians to provide the level of care that we propose. At the heart of this paper is also the calling for geriatricians to educate physicians who assume the role of the primary physician as well as subspecialists who may care for older patients. At very few academic institutions with Centers of Excellence, there may be cardiologists or oncologists who specialize in geriatric cardiology, geriatric oncology, or cardio-oncology. These experts may have additional insight or background in geriatrics to conduct a geriatric assessment and take into account issues specific to the older patient. However, at institutions lacking these hybrid practices or Centers of Excellence, education of primary physicians and other subspecialists by geriatricians can further facilitate GOC discussions, improve the overall care, and ensure that therapeutic options chosen and supportive care provided are synchronous with the older patient’s preferences and values.

## Conclusion

With advances in cancer and CV treatment, survival will improve and life expectancy will be extended. Not only will the initial diagnosis and treatment need attention, but physicians will also be faced with treating unintended consequences of these therapies. CV complications from cancer therapy, high burden of underlying CV disease, and age-related changes in physiology and metabolism represent the crossroads of geriatric cardiology and cardio-oncology. There is a paucity of data available for evidence-based recommendations in the cardio-oncologic care of older adult patients with cancer, but recent consensus statements for general management have been published to summarize available data and guide clinical care. Multiple professional organizations, research collaborations, and specialized centers have formed groups to address issues relating to cardio-oncologic care. Within the geri-cardio-onc collaborative framework, the geriatrician is well positioned to take an active leadership role in advocating for the patient, assisting with decision-making, and facilitating screening and long-term monitoring of CV complications. Further, education of primary physicians and subspecialists by geriatricians about geriatric principles may prove helpful in improving the overall quality of care in older adults.
